# Supplementary material for: Genomic epidemiology of Vibrio cholerae reveals the regional and global spread of two epidemic non-toxigenic lineages
Source: PLoS Negl Trop Dis. 2020 Feb 18;14(2):e0008046. doi: 10.1371/journal.pntd.0008046 (PMC7048298; doi:10.1371/journal.pntd.0008046)
Supplement: S2 Table — (PDF) [file pntd.0008046.s002.pdf]

S2 Table. The criteria used for clinical symptom classification.

	Mild	Moderate	Severe
Dehydration	<5%, child<br>2-3%, adult	5-10%, child<br>4-8%, adult	>10%, child<br>>8%, adult
Stool (day)	4-9	10-20	>20
Urine (day)	Normal	<400 ml	<50 ml
General appearance	Well, alert	Restless, irritable	Lethargic or unconscious
Voice	Nornal	Hoarseness	Hoarseness
Skin turgor	Instantaneous recoil	Non-instantaneous recoil	Very slow recoil (>2 s)
Lips	Normal/slightly dry	Dry	Dry
Eye socket, fontanelle	Normal/slightly Sinkage	Sinkage	Severe sinkage
Fingerprint	Normal	Shrivelled	Severe shrivelled
Muscle	Normal	Spasm	Severe spasm
Pulse	Normal	Rapid, low volume	Weak or absent
Systolic pressure	Normal	<9.33kPa (<70mmHg),child<br>12-9.33kPa (90-70mmHg), adult	<6.67kPa (<50mmHg), child<br><9.33kPa (<70mmHg), adult
