# Supplementary material for: Genomic epidemiology of Vibrio cholerae reveals the regional and global spread of two epidemic non-toxigenic lineages
Source: PLoS Negl Trop Dis. 2020 Feb 18;14(2):e0008046. doi: 10.1371/journal.pntd.0008046 (PMC7048298; doi:10.1371/journal.pntd.0008046)
Supplement: S3 Table — (PDF) [file pntd.0008046.s003.pdf]

S3 Table. Antimicrobial resistance of *ctxAB* negative, *tcpA* positive (CNTP) isolates.

	Resistant	Sensitive	Intermediate
Ampicillin	23% (18/80)	66% (53/80)	11% (9/80)
Amikacin	13% (10/80)	86% (69/80)	1% (1/80)
Streptomycin	5% (1/22)	95% (1/22)	0% (0/22)
Gentamicin	0% (0/80)	94% (75/80)	6% (5/80)
Tetracycline	1% (1/80)	99% (79/80)	0% (0/80)
Chloramphenicol	0% (0/80)	100% (80/80)	0% (0/80)
Trimethoprim-sulfamethoxazole (SXT)	4% (3/80)	95% (76/80)	1% (1/80)
Ciprofloxacin	0% (0/80)	100% (80/80)	0% (0/80)
Nalidixic acid	2% (0/58)	98% (0/58)	0% (0/58)
