# Supplementary material for: Genomic epidemiology of Vibrio cholerae reveals the regional and global spread of two epidemic non-toxigenic lineages
Source: PLoS Negl Trop Dis. 2020 Feb 18;14(2):e0008046. doi: 10.1371/journal.pntd.0008046 (PMC7048298; doi:10.1371/journal.pntd.0008046)
Supplement: S4 Table — (PDF) [file pntd.0008046.s004.pdf]

S4 Table. Summary of the Bayesian models used for BEAST analyses.

Model	Path sampling		Stepping stone sampling	
	Log marginal likelihood	Rank	Log marginal likelihood	Rank
Uncorrelated relaxed clock,Bayesion Skyline	-2172319.44 ± 0.17	1	-2172319.36 ± 0.05	1
Uncorrelated relaxed clock,Bayesion SkyGrid	-2172320.84 ± 0.87	2	-2172320.03 ± 1.20	2
Uncorrelated relaxed clock,Constant	-2172321.34 ± 0.25	3	-2172321.08 ± 0.07	3
Strict clock,Bayesion Skyline	-2172326.83 ± 1.31	4	-2172327.40 ± 1.40	5
Strict clock,Bayesion SkyGrid	-2172327.23 ± 1.51	5	-2172326.51 ± 1.71	4
Strict clock,Constant	-2172328.73 ± 0.43	6	-2172327.94 ± 0.33	6
Uncorrelated relaxed clock,GMRF Bayesion Skyride	-2172361.27 ± 3.92	7	-2172361.52 ± 3.83	7
Strict clock,GMRF Bayesion Skyride	-2172372.92 ± 1.37	8	-2172373.32 ± 1.72	8
