# Supplementary figures and images for: Genomic epidemiology of Vibrio cholerae reveals the regional and global spread of two epidemic non-toxigenic lineages
Source: PLoS Negl Trop Dis. 2020 Feb 18;14(2):e0008046. doi: 10.1371/journal.pntd.0008046 (PMC7048298; doi:10.1371/journal.pntd.0008046)

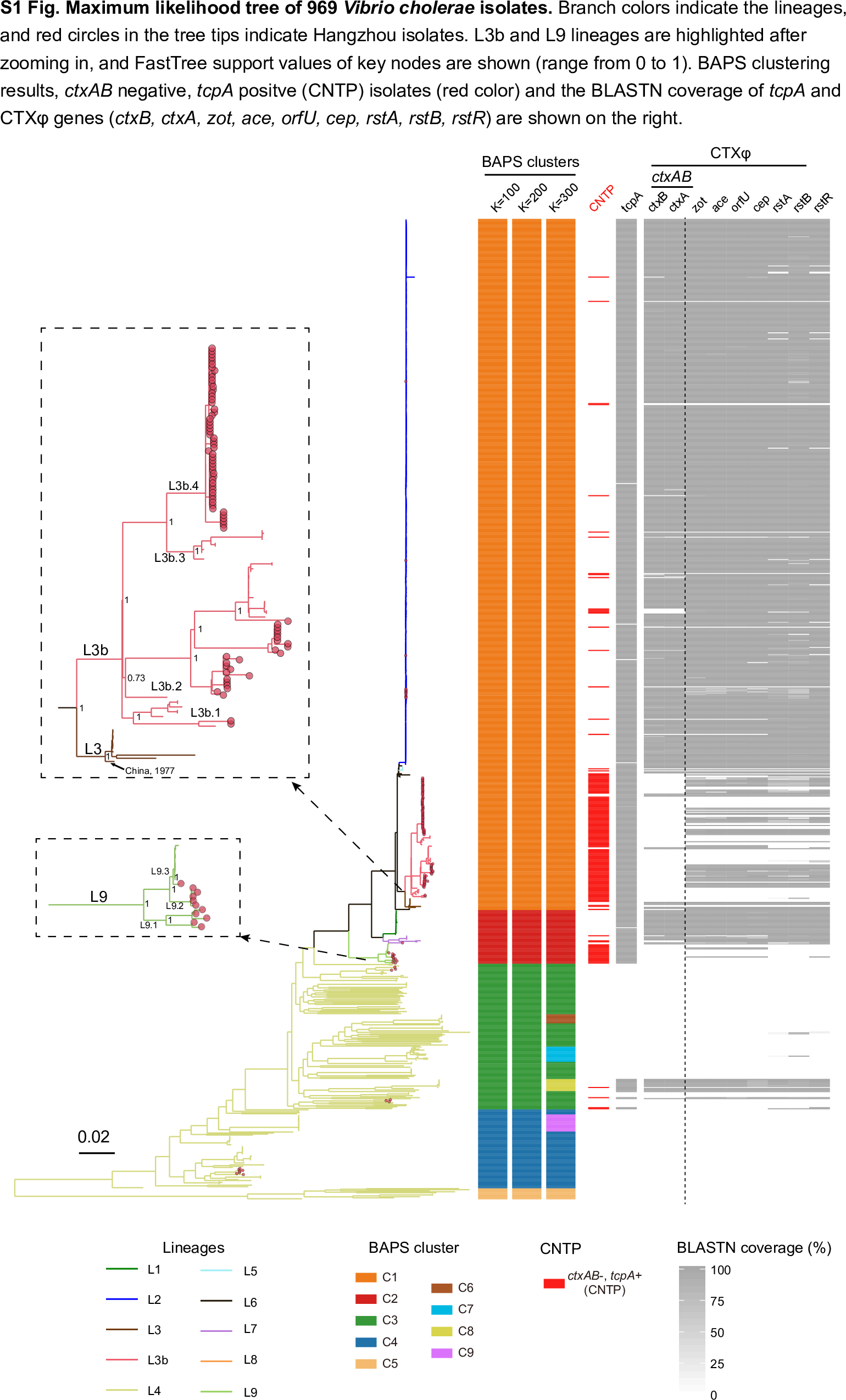

Supplement: S1 Fig — Branch colors indicate the lineages, and red circles in the tree tips indicate Hangzhou isolates. L3b and L9 lineages are highlighted after zooming in, and FastTree support values of key nodes are shown (range from 0 to 1). BAPS clustering results, ctxAB negative, tcpA positve (CNTP) isolates (red color) and the BLASTN coverage of tcpA and CTXφ genes (ctxB, ctxA, zot, ace, orfU, cep, rstA, rstB, rstR) are shown on the right. (TIF) [file pntd.0008046.s005.tif]

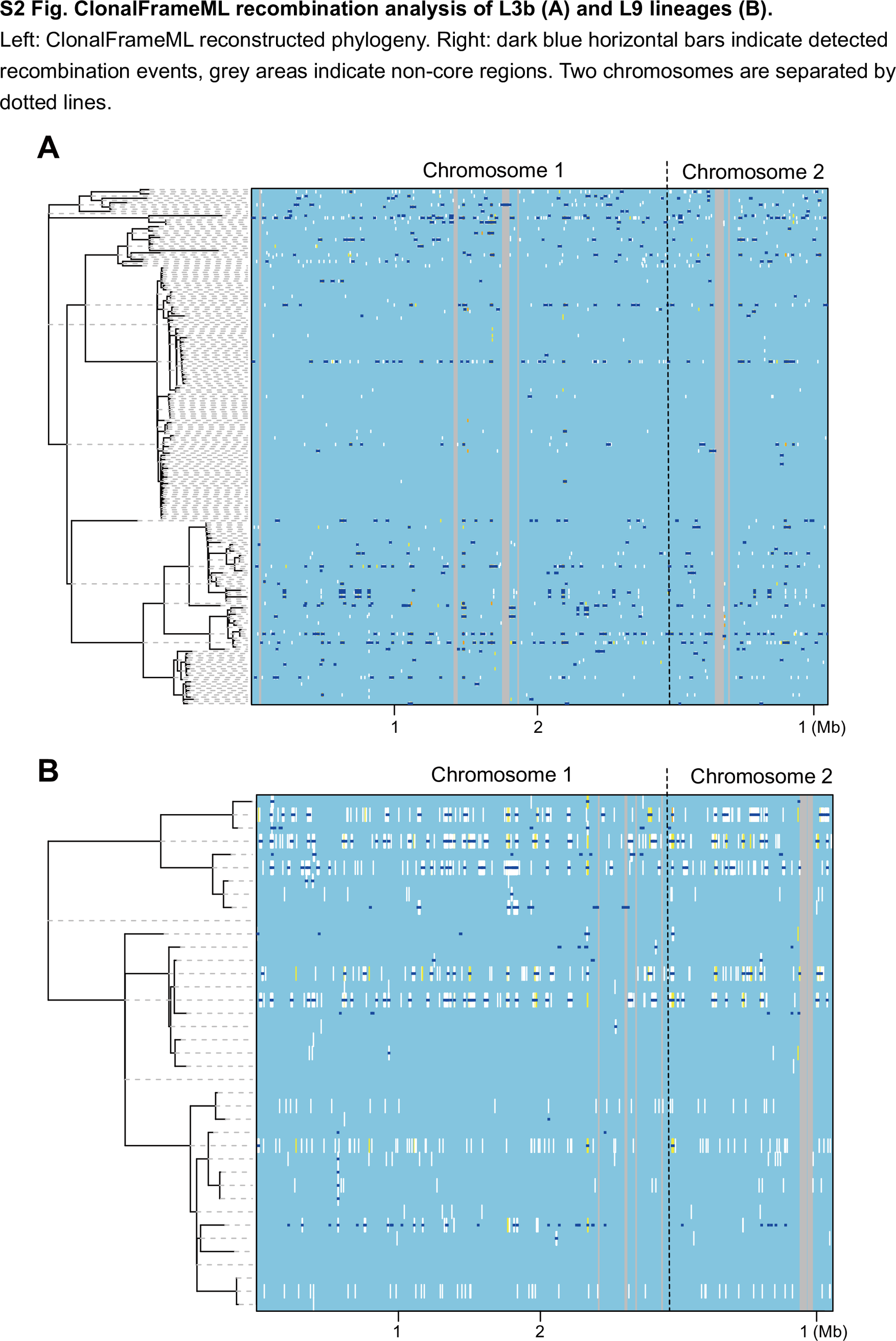

Supplement: S2 Fig — Left: ClonalFrameML reconstructed phylogeny. Right: dark blue horizontal bars indicate detected recombination events, grey areas indicate non-core regions. Two chromosomes are separated by dotted lines. (TIF) [file pntd.0008046.s006.tif]

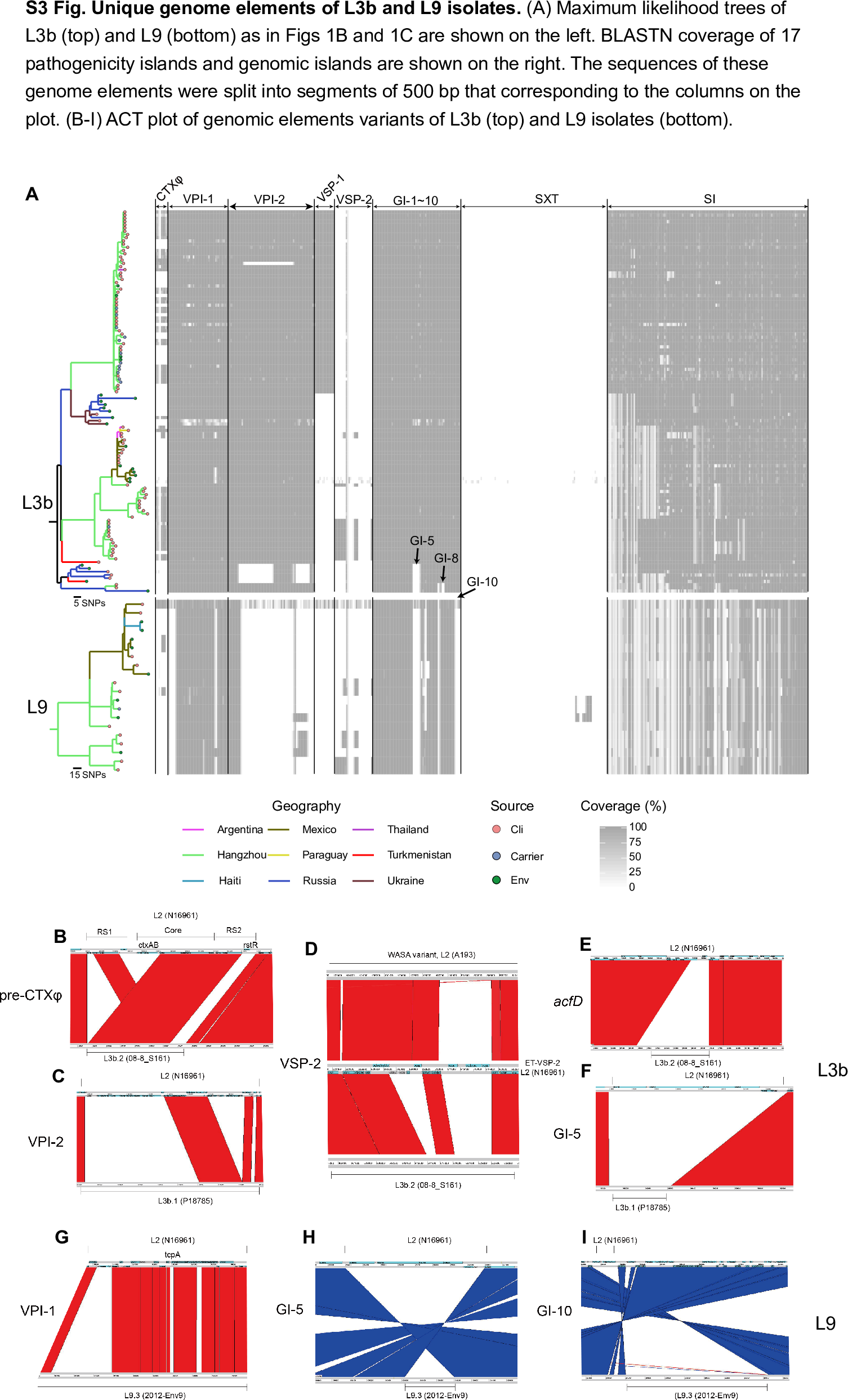

Supplement: S3 Fig — (A) Maximum likelihood trees of L3b (top) and L9 (bottom) as in Fig 1B and 1C are shown on the left. BLASTN coverage of 17 pathogenicity islands and genomic islands are shown on the right. The sequences of these genome elements were split into segments of 500 bp that corresponding to the columns on the plot. (B-I) ACT plot of genomic elements variants of L3b (top) and L9 isolates (bottom). (TIF) [file pntd.0008046.s007.tif]

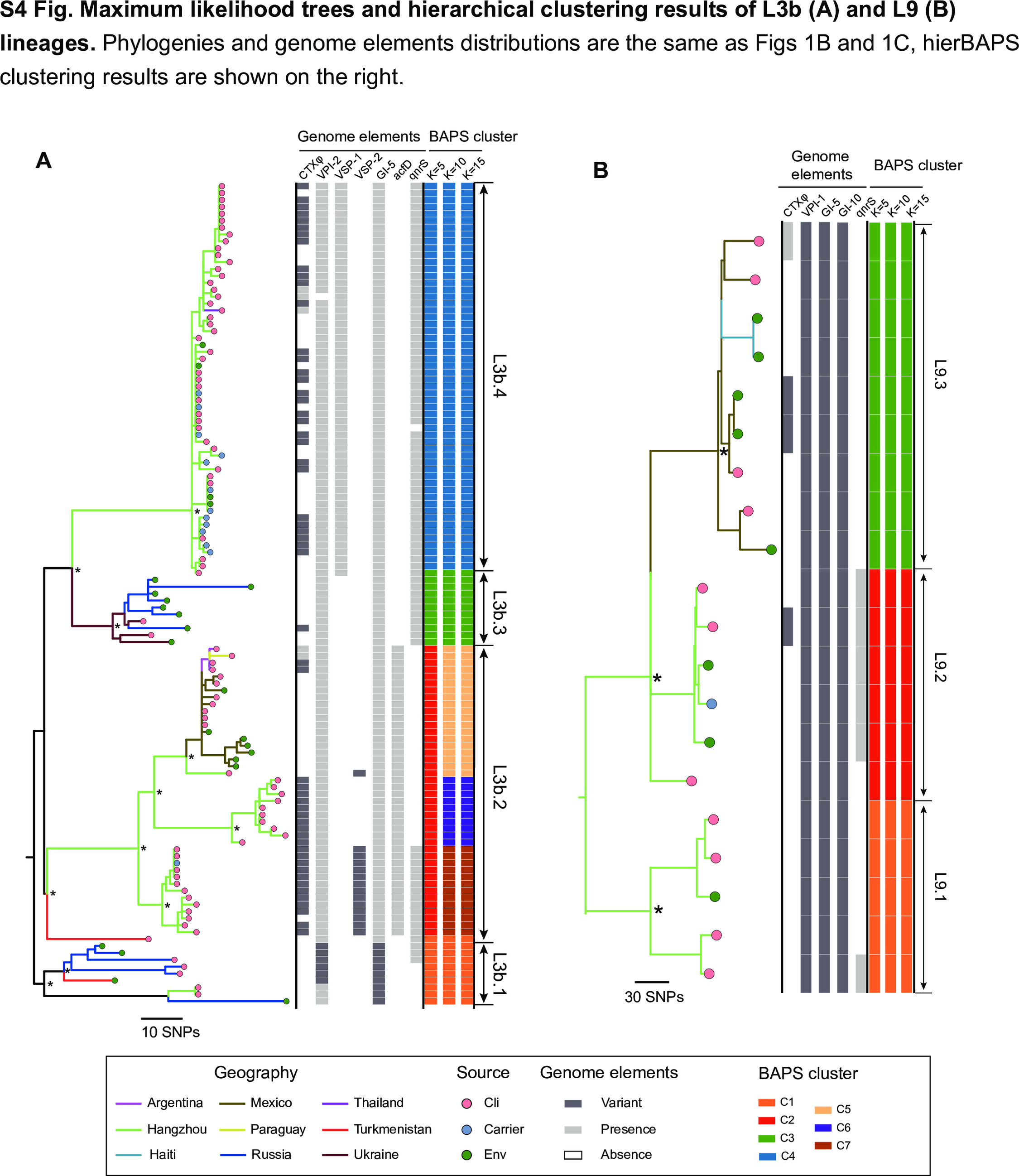

Supplement: S4 Fig — Phylogenies and genome elements distributions are the same as Fig 1B and 1C, hierBAPS clustering results are shown on the right. (TIF) [file pntd.0008046.s008.tif]

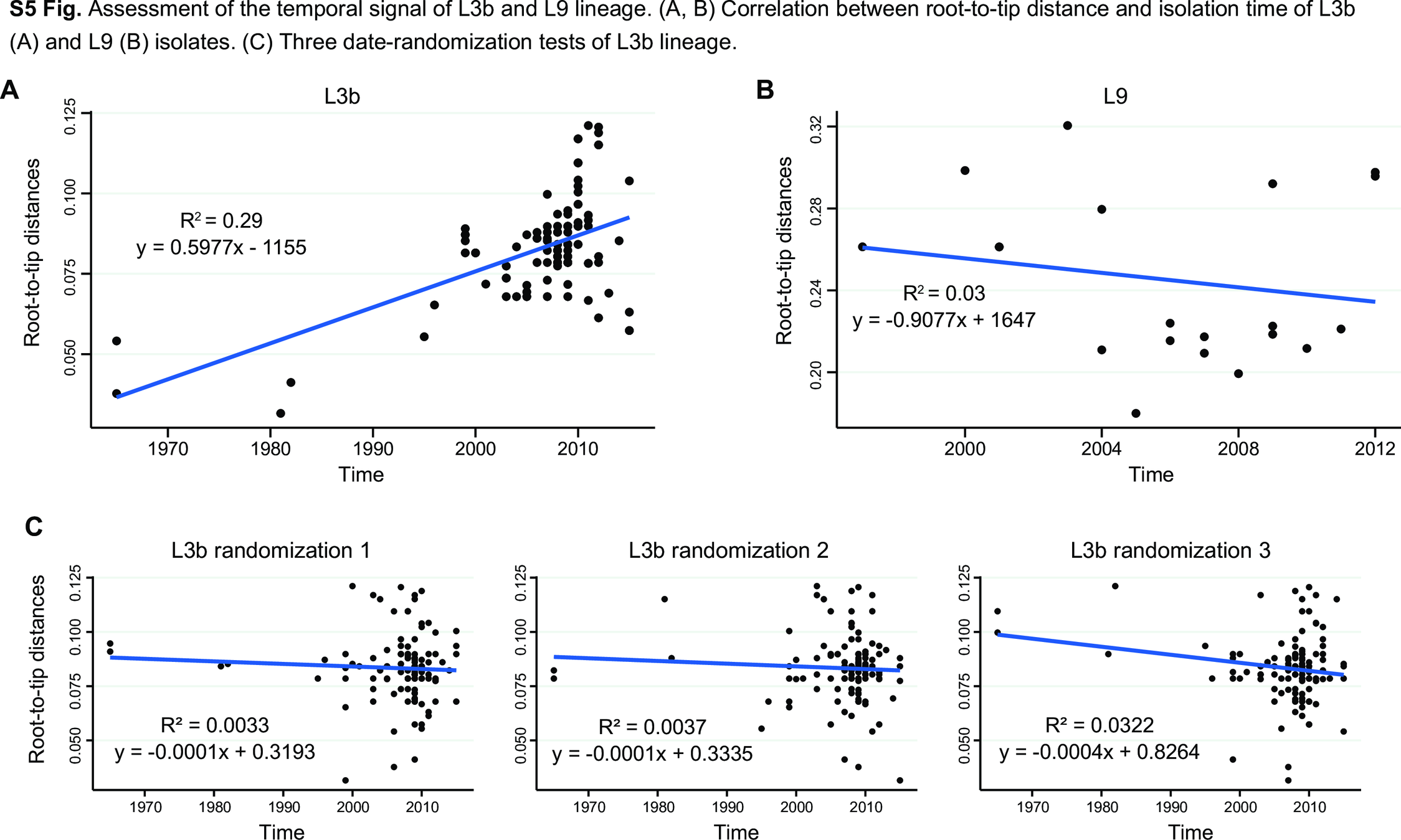

Supplement: S5 Fig — (A, B) Correlation between root-to-tip distance and isolation time of L3b and L9 (B) isolates. (C) Three date-randomization tests of L3b lineage. (TIF) [file pntd.0008046.s009.tif]

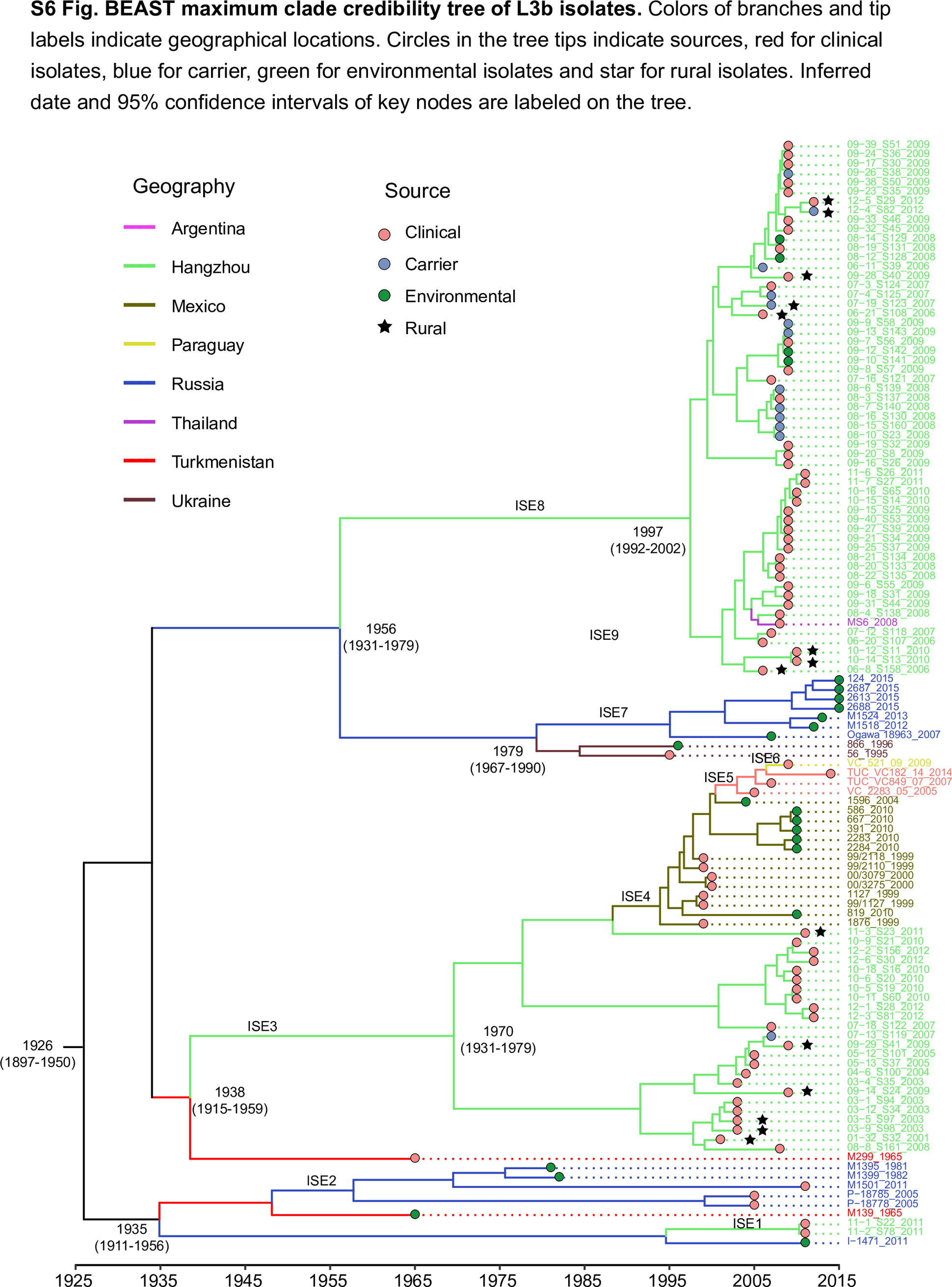

Supplement: S6 Fig — Colors of branches and tip labels indicate geographical locations. Circles in the tree tips indicate sources, red for clinical isolates, blue for carrier, green for environmental isolates and star for rural isolates. Inferred date and 95% confidence intervals of key nodes are labeled on the tree. (TIF) [file pntd.0008046.s010.tif]

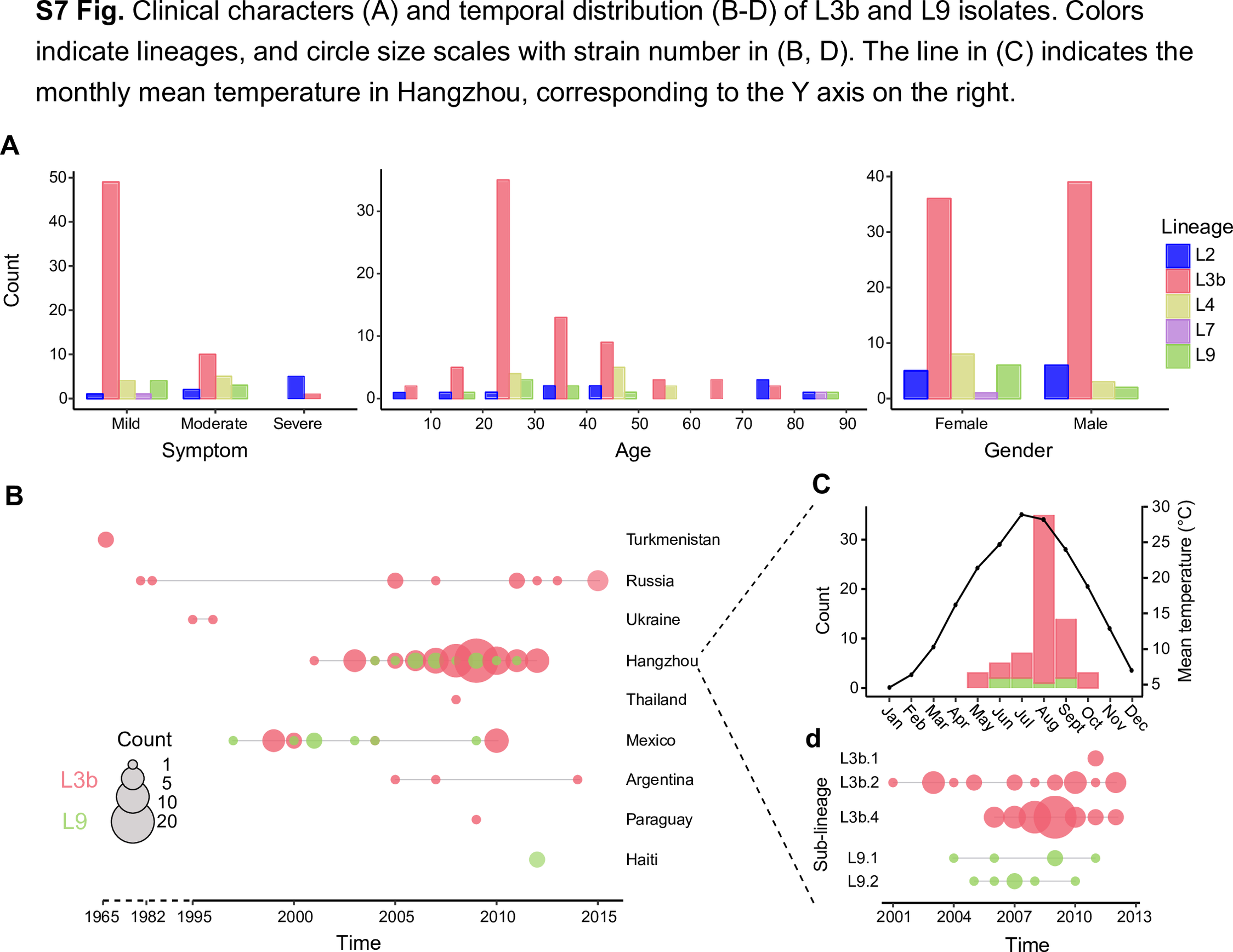

Supplement: S7 Fig — Colors indicate lineages, and circle size scales with strain number in (B, D). The line in (C) indicates the monthly mean temperature in Hangzhou, corresponding to the Y axis on the right. (TIF) [file pntd.0008046.s011.tif]

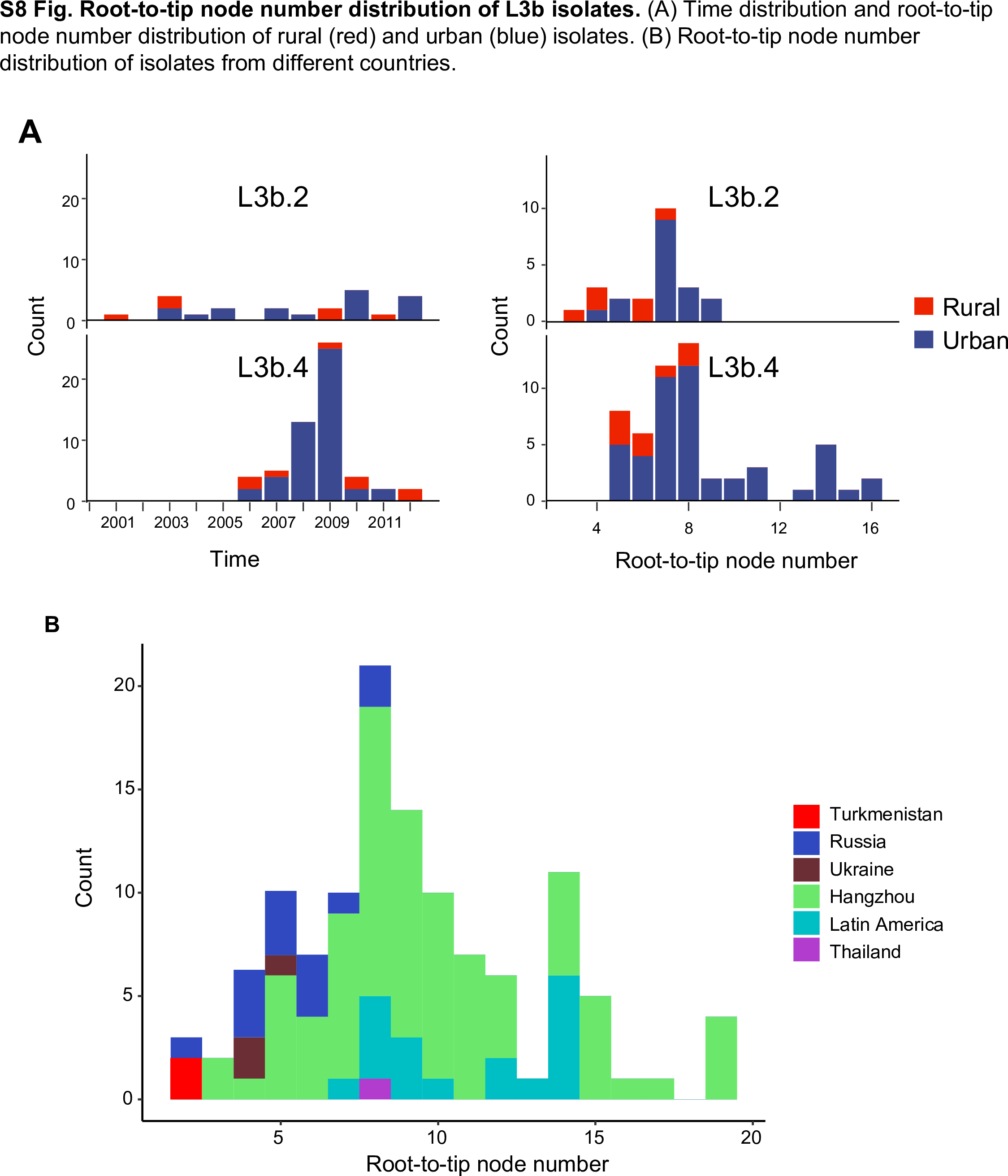

Supplement: S8 Fig — (A) Time distribution and root-to-tip node number distribution of rural (red) and urban (blue) isolates. (B) Root-to-tip node number distribution of isolates from different countries. (TIF) [file pntd.0008046.s012.tif]

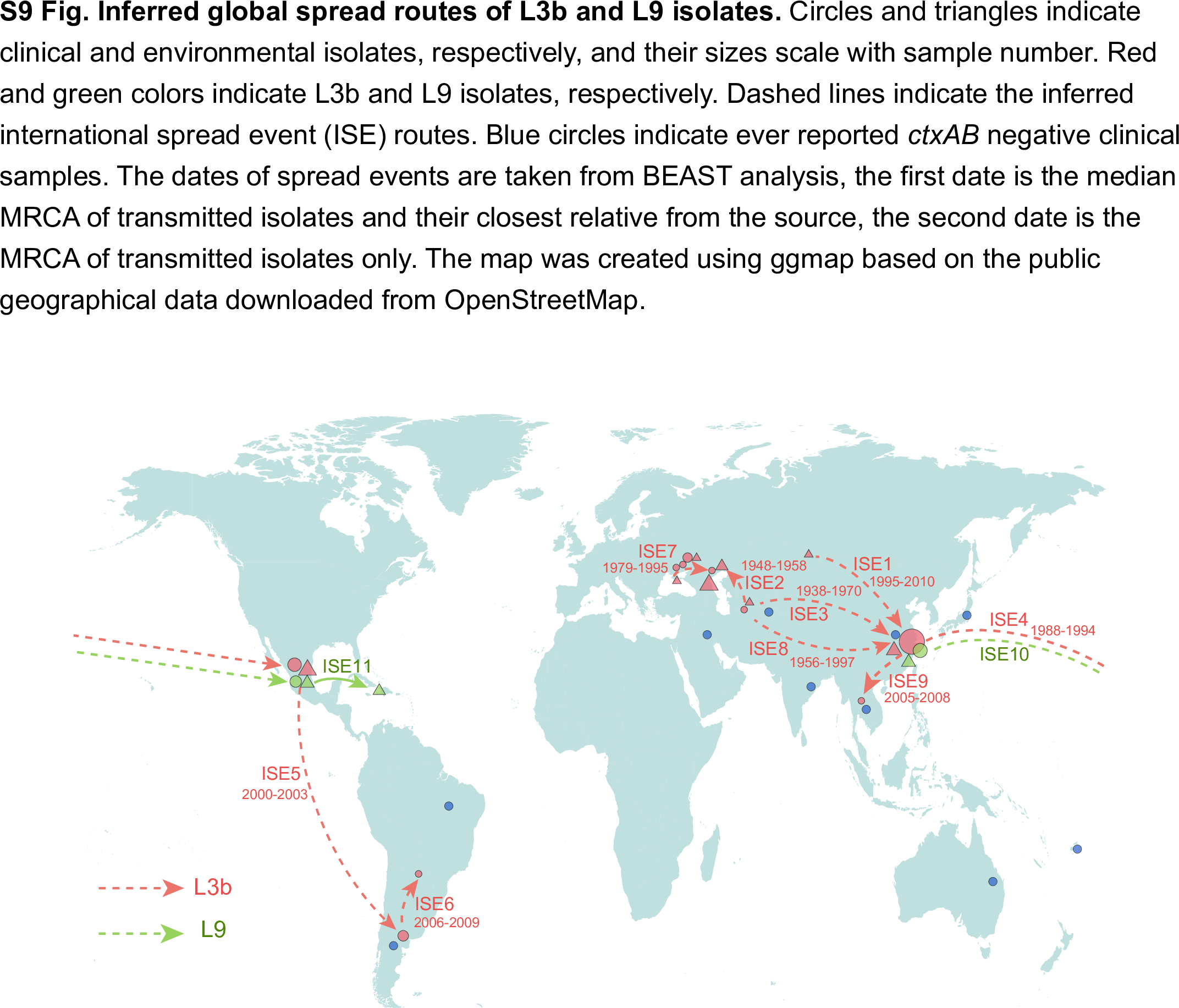

Supplement: S9 Fig — Circles and triangles indicate clinical and environmental isolates, respectively, and their sizes scale with sample number. Red and green colors indicate L3b and L9 isolates, respectively. Dashed lines indicate the inferred international spread event (ISE) routes. Blue circles indicate ever reported ctxAB negative clinical samples. The dates of spread events are taken from BEAST analysis, the first date is the median MRCA of transmitted isolates and their closest relative from the source, the second date is the MRCA of transmitted isolates only. The map was created using ggmap based on the public geographical data downloaded from OpenStreetMap. (TIF) [file pntd.0008046.s013.tif]
