# Supplementary material for: Genomic epidemiology of Vibrio cholerae reveals the regional and global spread of two epidemic non-toxigenic lineages
Source: PLoS Negl Trop Dis. 2020 Feb 18;14(2):e0008046. doi: 10.1371/journal.pntd.0008046 (PMC7048298; doi:10.1371/journal.pntd.0008046)
Supplement: Attachment — Submitted filename: 20191104-PNTD-R1.response letter.docx [file pntd.0008046.s014.docx]

**Tracking number: PNTD-D-19-00871**

**Title: “Genomic epidemiology of *Vibrio cholerae* reveals the regional and global transmission of two epidemic non-toxigenic lineages”**

**Responses to reviewers’ comments and suggestions:**

Reviewer's Responses to Questions

**Key Review Criteria Required for Acceptance?**
As you describe the new analyses required for acceptance, please consider the following:

**Methods**
-Are the objectives of the study clearly articulated with a clear testable hypothesis stated?
-Is the study design appropriate to address the stated objectives?
-Is the population clearly described and appropriate for the hypothesis being tested?
-Is the sample size sufficient to ensure adequate power to address the hypothesis being tested?
-Were correct statistical analysis used to support conclusions?
-Are there concerns about ethical or regulatory requirements being met?

**Reviewer #1:** (No Response)

**(Q1) Reviewer #2:** Overall, the aim of this study is clear and the sampling strategy — testing of all suspected cholera cases admitted to hospitals in China — was clear and appropriate. I have two relatively minor comments related to the selection and presentation of methods in the manuscript:

1- It would be helpful if the authors would present a few more details about how the samples were prepared for sequencing in the lab (the paragraph starting at line 154). Whole genome sequencing of cholera is still relatively new, and publishing detailed methods that were used to successfully produce whole genomes for analysis would be a helpful contribution to the field. In particular, there was no mention of the DNA extraction kit used and the DNA concentration used as input in the extraction protocol. Additionally, there are many Illumina protocols for preparing paired-end sequencing libraries (Nextera Flex, Nextera XT, etc.) - which one was used in this paper?
**Response:** We agree with the reviewer and have now provided the method for DNA extraction and sequencing library preparation in the revised manuscript as follows:

Lines 164-166: The DNA of Hangzhou isolates was extracted using DNeasy Blood & Tissue kit (QIAGEN), and subjected to paired-end library (average insert size of 350 bp) preparation using the NEBNext Ultra DNA Library Prep Kit (NEB).

**(Q2)** 2- It is unclear to me why the authors chose to present a neighbor-joining tree (rather than a maximum likelihood tree) in Figure 1a. Was there additional information gained from comparing the neighbor-joining tree produced in 1a to the maximum likelihood tree in Figure 1b? It should be computationally feasible to construct a maximum likelihood tree of the 969 isolates if using only SNP sites in the alignment, as stated in the Results.
**Response:** We chose the neighbor-joining (NJ) method because it is much faster than maximum likelihood method. Actually, we had firstly tried to construct a maximum likelihood tree of all the 969 isolates (257,757 SNPs) using RAxML, but the program was still not finished after more than a week. Considering the problem of limited computing resources, we chose NJ and obtained the NJ tree within one day.

Following the suggestion by the reviewer, we have now constructed a ML tree using FastTree (which is much faster than RAxML), to replace the previous NJ tree (Figure 1a and Figure S1). The topologies of the ML and NJ trees were very similar, thus different tree construction methods will not change our conclusion based on the previous NJ tree.

Figure 1a. Maximum likelihood tree of global 969 *V. cholerae* isolates. Branch colors indicate the lineages, and red circles in the tree tips indicate Hangzhou isolates. L2 lineage isolates were collapsed for visualization. Double slash indicates artificially shortened branch.

**(Q3)** Reviewer #3: I’m curious why you chose to assemble and then align using MUMmer, instead of mapping raw reads (which tend to be much more accurate in terms of calling variants) particularly for the within lineage analysis. See discussion here: https://doi.org/10.3389/fgene.2015.00235
**Response:** We agree with the reviewer that reads-mapping method tends to be more accurate for calling variants of within lineage isolates. However, as we cannot obtain the raw reads of the Russian isolates (important samples in this study), as well as many other worldwide isolates, we used assembly-based method to cover as many isolates as possible, especially for L3b isolates.

One problem of assembly-based method is that spurious SNPs can be included, because the coverage of each loci of an assembly was 1X. To solve this problem, we have mapped the clean reads of all the Hangzhou isolates back to the assemblies to assess the SNP quality, we only kept the SNPs supported by at least 10 reads and with the quality value > 30. In a recent study (Yoshimura *et al*., *Microbial Genomics* 2019), Yoshimura *et al*. reported that utilizing both assembly and mapping information can be more accurate than reads-mapping method, because the latter method can identify some false-positive SNPs located in “soft-clip regions” where many reads were soft-clipped (i.e. a read was partially not aligned because it was difficult to align the whole read to a single region).

After the reads-mapping process to flitter the low-quality SNPs, about <1% SNPs of Hangzhou L3b (139/27146, 0.5%) and L9 (169/19874, 0.85%) isolates were removed. If we hypothesize the qualities of public isolates were same as Hangzhou isolates, the small amount of low-quality SNPs will not lead to a bias which can influence the tree topology and the conclusion.

We have rewritten the SNP calling process in the revised manuscript as follows:

Lines 171-191: “SNPs were identified as previously described [11,33]. Firstly, the assemblies were aligned against the reference genome using MUMmer v3.1 [34], to generate the whole genome alignment and identify SNPs in the core genome (regions present in all isolates). Secondly, the clean sequencing reads of Hangzhou isolates were mapped to the assemblies to evaluate the SNP accuracy using SOAPaligner [35], only high-quality SNPs (supported by >10 reads, quality value >30) were kept in further analysis. The repetitive regions of the reference genome were identified using TRF v4 [36] and BLASTN search against self. SNPs in repetitive regions were excluded and only bi-allelic SNPs were used in further analysis. N16961 (NC_002505.1, NC_002506.1) was used as the reference sequence in the analysis of 969 genomes, a total of 562,172 SNPs (2.16 Mb core genome) were identified from the 969 isolates, which were used to construct the maximum likelihood tree using FastTree V2 [37]. Phylogenic analysis showed that the Hangzhou isolates belonged to two distinctive lineages, which were named L3b and L9 (Fig. 1a, Fig. S1).

The core regions of different genome sets are variable and this affects SNP calling and phylogenetic reconstruction. To obtain a high phylogenetic resolution, we re-selected reference sequences for L3b and L9 lineages to improve the whole genome alignments. Strain MS6 (NZ_AP014524.1, NZ_AP014525.1) was used as the reference sequences for L3b isolates, 40,533 SNPs (3.52 Mb core genome) were identified for all the 119 L3b isolates, 27,006 SNPs (3.68 Mb core genome) were identified for the 81 Hangzhou L3b isolates. Strain 2012Env-9 (NZ_CP012997.1, NZ_CP012998.1) was used as the reference sequences for L9 isolates, 18,937 SNPs (3.76 Mb core genome) were identified for all the 20 L9 isolates, 19,874 SNPs (3.83 Mb core genome) were identified for the 11 Hangzhou L9 isolates.”

**(Q4)** Line 151: The BioProject is not public yet on NCBI.
**Response:** The genome data of all the newly sequenced Hangzhou strains have been deposited in GenBank, we will release the data as soon as possible.

**(Q5)** Line 158: Can you provide the range of the number of contigs and sizes? What was the fold coverage of these genomes?
**Response:** The number of contigs of assemblies were 163 (46 – 495, > 500 bp), with the average size of 4.0 Mb (3.9 - 4.3), the average fold coverage of assembled genomes being 81 (54 - 96). We have now included this information in Material and Method section of the revised manuscript.

Lines 169-171: “The number of contigs and average size of assemblies were 163 (46 – 495, > 500 bp) and 4.0 Mb (3.9 - 4.3), with an average of 81-fold (54 - 96) depth for each *V. cholerae* genome.”

**(Q6)** It would be helpful to provide the alignment lengths for all of the mapping analyses performed, for instance the total number of SNPs in the mapping back to N16961.

Line 172: The 257,757 SNPs were called against which reference?

Line 172 -173: This is very confusing to follow. I would suggest re-arranging the sentence so that you report the SNPs per lineages together; ie 40,533 SNPs for the 119 L3b (27,006 SNPs for the 81 Hangzhou L3b isolates), etc…

When you are referring to ‘core genomes’ do you mean the MUMmer alignment against a reference? Core genome is more typically a term in a ‘pan-genome’ analysis to describe genes that are conserved in all or nearly all (~95%) genomes. 
**Response:** please see response to comment Q3 above and Line 171-191 in the revised manuscript. We have clarified the SNP calling process in the revised Material and Method part (Genome sequencing and variant calling section) and provided the reference genome, SNP number and core genome size of each dataset.

**(Q7)** What program was used to scale all of the ML trees into SNPs per site?
**Response:** The scale bar of phylogenies indicates nucleotide substitutions per site – that is, the number of substitutions throughout the length of the sequence. Multiplying this per-site number by the total number of sites in the input alignment we obtain scale bars in number of SNPs throughout the genomes (note that this calculation is valid because all genetic distances are relatively small so that repeat substitutions can be ignored).

**(Q8)** My biggest concern with the manuscript is how many sites were lost after applying ClonalFrameML to the datasets. For example, the alignment for L3b goes from 40,533 SNPs to 535 SNPs!! Yikes. As Xavier is an author on this paper, I would like to have his opinion on what is going on here.

Are the original SNPs distributed across the genome or do they mostly appear in clusters? If in clusters, perhaps looking at some of the reads mapped back to these regions might give an indication of what is going on.

**Response:** The recombination rate of L2 pandemic lineage (or 7PET lineage) was low (Mutreja *et al*., *Nature* 2011 and Domman *et al*., *Nature Genetics* 2018), Domman *et al*. calculated an overall *r/m* (ratio of effects of recombination and mutation) of 0.47 in the ClonalFrameML analysis of 7PET lineage. However, the recombination rates of L3b and L9 lineages are much higher. Based on our calculation, the overall *r/m* of L3b is 36.1, with ratio of recombination to mutation rate (*R/theta*) of 0.52, mean DNA import length (*delta*) of 4082 bp, and mean divergence of imported DNA (*nu*) of 0.017. The overall *r/m* of L9 lineage is 23.7, with the *R/theta*=0.67, *delta*=2716 bp, *nu*=0.013. This explains why so many SNPs were removed when considering only non-recombinant sites, as in the case of L3b noted by the reviewer. This situation is not uncommon when working with highly recombinant bacteria.

Although recombination was frequent in L3b and L9 lineage, after removing detected recombination regions, the core genome sizes still ranged from 1.56 Mb (L3b lineage) to 2.66 Mb (L9 lineage), we think the non-recombined SNPs in these regions are enough to generate robust phylogenies.

We have clarified this in the revised Material and Method part, and provided the ClonalFrameML result as the supplementary Figure S2:

Lines 194- 210: We used ClonalFrameML [38] to infer the recombination events in L3b and L9 lineages as previously described [39]. The input maximum likelihood trees were constructed using FastTree V2 [37] based on the whole genome alignments, non-core sites were ignored during the calculation. ClonalFrameML analysis revealed that the recombination rates of L3b and L9 lineages were much higher than that of L2 pandemic lineage (Fig. S2). The ratio of effects of recombination and mutation (*r/m*) of L3b lineage was 36.1, with ratio of recombination to mutation rate (*R/theta*) of 0.52, mean DNA import length (*delta*) of 4082 bp, and mean divergence of imported DNA (*nu*) of 0.017. The *r/m* of L9 lineage was 23.7, with *R/theta*=0.67, *delta*=2716 bp and *nu*=0.013. In contrast, the recombination rate of L2 pandemic lineage was much lower, with *r/m* previously reported to be 0.47 [10].

SNPs located in inferred recombined regions were excluded prior to further phylogenetic analysis. After removing the recombinant SNPs, 535 SNPs (1.56 Mb non-recombined core genome) for L3b isolates, 662 SNPs (2.23 Mb non-recombined core genome) for Hangzhou L3b isolates, 509 SNPs (2.66 Mb non-recombined core genome) for L9 isolates and 448 SNPs (2.72 Mb non-recombined core genome) for Hangzhou L9 isolates were used to construct the maximum likelihood trees using RAxML v8 [40] under the GTRGAMMA model with 100 bootstraps (Fig. 1b, c). All the phylogenetic trees were visualized using ggtree v1.16 [41].

**(Q9)** Using the core-gene alignment from the Latin American paper, two strains (TUC_VC182 & 2284) within MX-2 have 749 SNPs between them. Looking at your Fig 1b, the scale bar would indicate there are less than 10-20 SNPs between any MX-2 isolates. It would be helpful to see the pre-CFML sub-trees of L3b and L9 to judge relatedness.
**Response:** In the pre-CFML sub-trees, the SNP distance between two MX-2 isolates (TUC_VC182 & 2284) was 2,294, which is 3 times larger than the number based on core-gene alignment of Latin American paper. This difference should be caused by different core genome sizes, the core genome size of 119 L3b isolates was 3.57 Mb, which is also 3 times larger than that in the Latin American paper (1.01 Mb, 2,105 core genes). The SNP density of the two datasets was quite similar (0.64 vs. 0.74 SNPs per Kb).

**(Q10)** I would suggest that the authors use some sort of clustering algorithm (such as hierarchical BAPS) to guide how they designate lineages. It seems that there is tremendous diversity in just the L3b clade, to the point where I would be very hesitant to label them the same (sub)lineage.
**Response:** We thank the reviewer for the constructive suggestion. We perform clustering analysis of L3b and L9 lineages using hierBAPS based on the non-recombined SNP alignment, with the maximum number of populations (*K*) of 5, 10 and 15. L3b lineage isolates were split into four or six hierBAPS clusters, and L9 lineage isolates were split into 3 clusters which are consistent with our assignment (Figure S4, below). For L3b lineage, hierBAPS cluster C2 (*K* =5) were split into three clusters (C5-C7) when *K* was set to 10 and 15, however, in the phylogenetic tree, it was clear that isolates of C5-C7 have a common ancestor, which were supported by a long common branch with a high bootstrap value (100). Thus, we prefer to assign C5-C7 into one cluster, and designate L3b lineage into four sub-lineages, L3b.1-L3b.4. Additionally, one hirBAPS cluster C1 isolate M229, clustered together with L3.2 (C2 or C5-7) isolates in the phylogenetic tree, which were supported by a high bootstrap value (100), we still prefer the clustering result of phylogeny and assigned it into L3b.2 sub-lineage. We have provided the BAPS result as the supplementary Figure S4 and clarified this in the revised Material and Method part as follows:

Line 235-243: HierBAPS was then run on the non-recombined SNPs of L3b and L9 lineages, with the maximum *K* of 5, 10 and 15. L3b lineage isolates were split into four or six hierBAPS clusters, and L9 lineage isolates were split into 3 clusters which are named L9.1-L9.3 sub-lineages (Fig. S4). For L3b lineage, hierBAPS cluster C2 (*K* =5) were split into three clusters (C5-C7) when *K* was set to 10 and 15, however, in the phylogenetic tree, it was clear that isolates of C5-C7 have a common ancestor, which were supported by a long common branch with a high bootstrap value (100). Combining the hierBAPS clustering and phylogenic tree, we designated L3b lineage into four sub-lineages, L3b.1-L3b.4. Additionally, one hierBAPS cluster C1 isolate M229, clustered together with L3.2 (C2 or C5-7) isolates in the phylogenetic tree, which were supported by a high bootstrap value (100), we assigned it into L3.2 sub-lineage.

**(Q11)** For the root-to-tip distances, can you please load the L3b tree into TempEst and provide me the plot showing the ancestor traces please? This can help in identifying possible isolates that might be problematic. See here for further details: http://beast.community/tempest_tutorial. It does seem that if you did not include the four L3b isolates sampled prior to 1990, there might be very little signal.

Given that the R^2 values are not very good for the root to tip data, I’m going to be a pain and ask that the authors do a date randomization test to further confirm that the temporal signal in the data is real.

**Response:** We thank the reviewer for the constructive suggestions. After loading the L3b tree into TempEst and showing the ancestor traces, we can see many points deviated the regression line. If we ignore the isolates sampled before 1990, the correlation becomes much worse (*R^2^* =0.08). To test the temporal signal, we performed three date-randomization tests using R package TipDatingBeast. The correlation coefficients of date-randomization tests were much smaller, and the correlations were not significant, indicating the temporal signal of L3b lineage should be real (Figure S5, below). We have added the date-randomization tests as Figure S5c, and classified these in the revised manuscript as follows:

Line 246-249: “We investigated the temporal signal of L3b and L9 lineages using TempEst v1.5 [46], by calculating the linear regression between root-to-tip distance and isolation date. Three date-randomization tests were performed using TipDatingBeast [47], and we found a significant temporal signal in the L3b lineage (Fig. S5).”

Figure #. TempEst result of L3b lineage showing ancestor traces.

**(Q12)** Did you only run a single MCMC chain for each model in BEAST? Typically, one runs a minimum of three as to avoid getting stuck in local maxima. It might make sense to test other tree priors, such as non-parametric models like the Skyride or Skygrid as it may be that the L3b population has not remained stable over time. 

For the BEAST analysis, did the authors correct for constant sites by adding in these values to the XML files as described here: https://groups.google.com/forum/#!searchin/beast-users/SNPs/beast-users/V5vRghILMfw/jMtC_DwS5EYJ
**Response:** We thank the reviewer for the constructive suggestions about the usage of BEAST. In the previous analysis, we indeed ran a single MCMC chain and did not correct for constant size. As suggested by the reviewer, we have now performed three independent MCMC chains for each model, corrected for constant sites and tried three non-parametric models (SkyGrid, Skyride and Skyline) in the new analysis. Both path sampling and stepping-stone sampling indicate the best fit was an uncorrelated relaxed clock with a Bayesian skyline coalescent tree (Table S4, below).

We ran three independent Markov chain Monte Carlo chains and combined them to generate the maximum clade credibility trees (Figure S6, below) and date the spread events. The estimated date of spread event based on new analysis were similar to previous results. Based on new analysis, the clock rate of L3b lineage was 3.73×10^-7^ substitutions per site per year, which is still lower than that of L2 pandemic lineage. We have classified this in the revised manuscript as follows:

Line 249-263: “The 118 L3b isolates with known isolation dates were further used in BEAST v1.10 [48] to date the common ancestors. 535 non-recombinant SNPs were included in this analysis, using the GTR + Γ substitution model, isolation years for tip date calibration and constant sites correction. We tested eight combinations of molecular clock and tree models to identify the best fit, three independent chains for each combination were run to ensure convergence. Both path sampling and stepping-stone sampling indicate the best fit was an uncorrelated relaxed clock with a Bayesian skyline coalescent tree (Table S4), which were used as the priors of further analysis. We ran three independent Markov chain Monte Carlo chains over 100 million steps, sampling every 2,000 steps. Tracer v1.7 [49] was used to read the BEAST outputs and calculate the effective sample size (ESS) values, and the ESS of parameters of all three runs were greater than 200. The tree estimates of three runs were combined using LogCombiner v1.10 and were used to generate the maximum clade credibility trees (Fig. S6) using TreeAnnotator v1.10, with the first 10% of states removed as burn-in. The BEAST estimated molecular clock of L3b lineage strains was 3.73×10^-7^ substitutions per site per year, with 95% confidence interval (CI) 2.78-4.72×10^-7^, which is lower than the previously estimated mutation rate of the seventh pandemic group: 6.1×10^-7^ (95% CI: 5.7-6.4×10^-7^) substitutions per site per year [9].”

**Results**
-Does the analysis presented match the analysis plan?
-Are the results clearly and completely presented?
-Are the figures (Tables, Images) of sufficient quality for clarity?

Reviewer #1: (No Response)

**(Q13)** Reviewer #2: Figures 1-3 were clear, easy to understand, and supported the conclusions described in the text. While Figure 4 is clear and provides a nice visual explanation of the conclusions described in “Evidence for the global spread of L3b and L9 lineages”, I am generally uncomfortable with the visualization of arrows showing movement based on a small number of samples. The results are described nicely and with the appropriate caution (i.e. lines 344-345) but I find the solid line arrows on the map potentially misleading. For example, the evidence presented that Mexico strains descended from Hangzhou strains is convincing, but how do you know the transmission was direct from Hangzhou to Mexico, without a (possibly unrecorded or minimally symptomatic) intermediate? I would suggest moving this figure to the supplemental material, to avoid continuing the pattern in the cholera field of presenting tentative transmission events as hard facts.
**Response:** we agree with the reviewer that we should be more careful for the arrows, leaving the arrows for transmission events supported by hard facts. As suggested by the reviewer, we have moved previous figure 4 to supplementary Figure S9 (below). As these two lineages were most likely to be neglected by the epidemiology surveillance, we don’t have the hard facts supporting the transmission, thus, we have changed all the solid lines into dotted lines, indicating possible transmissions without clear epidemiological evidences.

**(Q14)** Another small comment: it would be helpful if the authors could provide the number of SNPs in their full SNP alignments, both to add context to their comments about the similarity between human and environmental isolates (line 308) and to allow for comparison to previous studies of global cholera transmission. This could be provided in methods (around line 172) or when describing the results (line 221).
**Response:** We admit this information is important, in the revised manuscript, we have rewritten the SNP calling process to classify the number of SNPs of each dataset, please refer to the response to comment Q3.

**(Q15)** Reviewer #3: Of the 850 genomes included in the study, 540 of them were from 7PET (L2). This seems unnecessary as there was no analysis of these strains in the paper.
**Response:** We agree with the reviewer that including high fraction of 7PET isolates in the study seems unnecessary. However, we included all the publicly available assembled genomes for comparison, whether or not 7PET isolates, we did not know the lineage information before we analyzed them. After lineage assignment, we focused on L3b and L9 lineage and ignored the L2 pandemic lineage.

**(Q16)** Line 220-230: How were lineages assigned and what criteria was used to designate or differentiate lineages? Same question for the sub-lineages. It seems L4 is a catch all for many different lineages, for instance there are 6 lineages from the Latin American paper that are within what you call L4. 
**Response:** We agree with the reviewer that a more data-driven method is needed to defined the lineages, as suggested by the reviewer, we performed clustering analysis for all the 969 isolates using hierBAPS, with the maximum number of populations (*K*) of 100, 200 and 300. 969 isolates were clustered into five (*K*=100 and 200) or nine (*K*=300) lineages (Figure S1). Lineage L2, L3, L3b, L5, L6 and L8 were clustered into hierBAPS cluster C1, lineage L1, L7 and L9 were clustered into hierBAPS cluster C2, and Lineage L4 were split into three or seven hierBAPS clusters. HierBAPS clusters generally contain more isolates than that of L1-L9, and no CNTP isolates specific clusters were identified. To focus on the CNTP isolates and keep the unity to previous study (Mutreja *et al*., *Nature* 2011), we would like to use the designation of lineage L1-L9 in this study. In Figure S1, we can see that most L4 lineage isolates do not carry any common virulence factors, and most of them were environmental isolates, clustering them together seems reasonable in an epidemiological sense. we have now provided BAPS clustering results in the supplement (Figure S1) and in the main text we now make explicit that our purpose is to focus on L3b and L9, and L4 lineage can be split into more BAPS clusters as follows:

Line 225-233: “We performed clustering analysis for all the 969 isolates, L3b and L9 lineage isolates using the hierarchical Bayesian Analysis of Population Structure (hierBAPS) [45]. Firstly, hierBAPS was run on the 562,172 SNPs of 969 isolates with the maximum number of populations (*K*) of 100, 200 and 300. 969 isolates were clustered into five (*K*=100 and 200) or nine (*K*=300) lineages (Fig. S1). Lineage L2, L3, L3b, L5, L6 and L8 were clustered into hierBAPS cluster C1, lineage L1, L7 and L9 were clustered into hierBAPS cluster C2, and lineage L4 were split into three or seven hierBAPS clusters. HierBAPS clusters generally contain more isolates than that of L1-L9, and no CNTP isolates specific clusters were identified. To focus on the CNTP isolates and keep the unity to previous study [5], we used the designation of lineage L1-L9 in this study.

**(Q17)** Line 244: Was the one case that showed classic rice water stool CPTP?
**Response:** It’s a CNTP isolate, we have revised the sentence to include this information as follows:

Line 293: “only one case **caused by CNTP isolate** showed typical “rice-water” stool, we…

**(Q18)** Line 300: Are these tip-to-root node distances based off of the recomb-free SNP alignment only or the pre-CFML alignment?
**Response:** All the tip-to-root node distances were calculated based on recomb-free SNP alignment.

**(Q19)** Line 321-323: Awkward sentence, consider re-wording. 
**Response:** We have rewritten these sentences to make it clear as follows:

Line 372-374: “They were isolated from patients (48%) or environment (52%, water and food) from 1965 to 2015, during the diarrheal/cholera outbreaks or by routine surveillance”

**(Q20)** Global spread of lineages: I think the authors go too far in here in associating these lineages to global spread. As stated above, the loss of so many sites made these strains artificially appear very similar to those sampled in LA and elsewhere. Again, looking that the NJ tree in FigS1, there is more diversity within L3b than seen between L7 and L1. Also true for diversity seen between any lineages at the top of the tree (L5, L8, L6 & L2). There is one long branching isolate from China that is at the base of the MX-2 lineage (your L3b.2), which does not appear to have high bootstrap support. It does seem that ITE9 is plausible, likely due to shipment of contaminated food products, as this strain sits well within those from Hangzhou. 
**Response:** We admit that sampling bias is likely to exist for this analysis, thus we have written in a less assertive way the description of the spread routes, with the aim of highlighting that global spread of L3b and L9 lineages do exist. We here used the subtitle of “evidence of global spread”. In addition, as suggested by Reviewer 2, we have now moved previous figure 4 into supplementary files (Figure S9) to further classify this.

Specifically, the high genetic diversity of L3b lineages in Figure S1 should be the result of high recombination rate, as we stated above. After removing the recombination sites, only 535 SNPs located in 1.56 Mb non-recombined core genome (one SNP per 2.9 Kb) were identified, the SNP density is even smaller than that of L2 pandemic isolates (9300 SNPs in 3.97 Mb core genome, one SNP per 435 bp in Weill et al, *Science* 2017). In addition, the high genetic diversity of L3b lineages could also indicate the time of origin and existence was longer. We dated the MRCA of L3b lineage in 1926 (95% CI, 1897-1950), which is older that of L2 lineage (1952, Mutreja *et al*., *Nature* 2011).

The close relationship between the China isolate and MX-2 isolates was supported by a high bootstrap value, before (Figure S1, FastTree support value = 1) or after (Figure 1b, bootstrap value = 100) removing recombined SNPs. In previous Figure S1, we only showed the bootstraps of key nodes, the bootstrap value of this node was not labeled.

We can only know MS6 was a Thailand isolate which was isolated from a patient in an outbreak, which could be caused by the contamination of shipped food. Anyway, this also is an evidence of the global spread of L3b.

General questions:
**(Q21)** Do the authors think that there is secondary transmission (ie person to person)? Or rather is this a point source / intermittent common source of food borne illness? 
I bring this up as the words ‘transmission’ and ‘introduction’ are used in the manuscript. Perhaps I have misunderstood their meaning, but I take this to mean human to human transmission, which I am not convinced is the case here. 
**Response:** We think this is a point source/intermittent common source of food borne illness, such as the outbreak in the restaurant (Line 360-368), which was confirmed by the epidemiological investigation and the WGS analysis. We discuss this in the previous version of manuscript, now we further revised the text to highlight this (Line 450-454).

At the moment, we do not have any evidence supporting human to human transmission. To make this clear, we have rewritten the manuscript to avoid the use of the word “transmission” where it could be misunderstood as human-human transmission.

Title: “Genomic epidemiology of *Vibrio cholerae* reveals the regional and global **spread** of two epidemic non-toxigenic lineages”

Line 29: “the **spread** route of the L3b lineage was…”

Line 30-31: “Both lineages had been previously reported as causing local **endemic disease** in Latin America…”

Line 33: “Other **spread** events, including from China to Thailand…”

Line 48-49: “…and a **spread** pattern from rural aquafarms to the urban areas was observed…”

Line 341: “The local **epidemiology** of L3b and L9 lineages in Hangzhou”

Line 377-378: “we only aimed to construct the initial global picture of the **epidemiology** of these two lineages.”

Line 380: “we observed that at least nine international **spread** events (ISE)”

Line 395: “…the **spread** direction of L3b lineage was…”

Line 411: “The global **spread** route of L3b strains are…”

Line 419: “…detect a significant temporal signal and estimate **dates…**”

Line 645: “Figure 2. Local **spread** of L3b and L9 lineages in Hangzhou.”

**(Q22)** Figure 1: Need to state that the b & c panels are ML trees from alignment post CFML. Also, the branch color for Paraguay is very difficult to see in the tree. You might consider changing this to a darker color. 
**Response:** We have now clarified this in the figure legend and changed the color for Paraguay.

Figure 1 legend. Phylogeny of global 969 *V. cholerae* isolates (a), L3b (b) and L9 (c) isolates. (a) Maximum likelihood tree of global 969 *V. cholerae* isolates. Branch colors indicate the lineages, and red circles in the tree tips indicate Hangzhou isolates. L2 lineage isolates were collapsed for visualization. (b,c) Maximum likelihood trees of L3b (b) and L9 (c) lineages **based on non-recombined SNPs.** L3b.1 and L9.1 isolates were used to root the trees based on Fig. 1a and Fig. S1. Branch colors indicate the geographical locations, and circles in the tree tips indicate the sources, red for clinical (Cli) isolates, blue for carrier and green for environmental (Env) isolates.

**(Q23)** I would suggest moving Figure 2 to the supplement as well as panel D from Figure 3 -- maybe add this to Fig S5. 
**Response:** We have now moved Figure 2 into supplementary Figure S7, and move panel D of previous Figure 3 into Figure S8a.

**(Q24)** Fig S1: Can you please label on the tree the other L3b sub-lineages as you have done with L3B.1.
**Response:** We have labeled L3b.1 to L3b.4 in the revised Figure S1.
--------------------

**Conclusions**
-Are the conclusions supported by the data presented?
-Are the limitations of analysis clearly described?
-Do the authors discuss how these data can be helpful to advance our understanding of the topic under study?
-Is public health relevance addressed?

Reviewer #1: (No Response)

Reviewer #2: The conclusions are in general clear and well supported/caveated. However, there are two conclusions I feel are not entirely supported by the data that I would suggest the authors address:

**(Q25)** 1- In line 254, the authors claim the incidence rate of L3b cases revealed temporal fluctuation. However, I would argue that it’s possible that they happened to see an outbreak of this lineage during the sampling time frame, and that it’s possible the other lineage caused an outbreak outside of the 2001-2012 time period. It may be more appropriate to state that an outbreak was observed for L3b cases, and not try to draw larger conclusions from this observation.
**Response:** Thank you for pointing this out. We have followed the suggestion and change it into an outbreak as follows:

Line 301-306: “Different temporal patterns of incidence were observed. The L9 lineage only caused sporadic cases uniformly distributed over time, **whereas an outbreak was observed for L3b cases in 2009. More specifically, the outbreak was mainly caused by the sub-lineage L3b.4**, which first appeared in Hangzhou in 2006, after which the number of reported cases rapidly increased until its peak in 2009, and then quickly decreased in 2010 (Fig. S7d), without any specific infectious disease control measure having been taken.”

**(Q26)** 2- In line 294, the authors comment that it is interesting to see that isolates from the southeast rural area belonged to L3b.2 and ones from the northeast were L3b.4. I don’t find this particularly surprising or remarkable, since it would be expected that lineages would cluster somewhat by geography. I have no problem with the authors describing their observations, I just don’t find it particularly noteworthy.
**Response:** Compared to the distribution in the urban areas where all the lineages mixed together, the rural isolates are clustered by geography, we think this is interesting to point out, because it indicates different type of causative agent for the cases in rural, and is helpful to infer the origin of sub-lineages. Thus, we would like to revise the sentence to make it more “useful” as follows:

Line 343-347: “The 81 L3b isolates showed a broad geographical distribution, which covered both urban area and rural districts, whereas ten L9 isolates originated almost exclusively from the urban area (Fig. 2a). **Compared to the distribution in the urban areas where all the sub-lineages mixed together, the rural isolates are clustered by geography, all the isolates from the southeast rural area belonged to L3b.2, and in contrast most northeast rural isolates were L3b.4 isolates.”**

**(Q27)** Reviewer #3: This is nice work describing outbreaks caused by non-pandemic VC lineages. I agree with the authors that we need a much better sampling of non-pandemic VC. 

The link to sea turtles as potential transport mechanisms seems a bit too tenuous at this stage.
**Response:** We agree with the reviewer that we have no clear evidence supporting the transport mechanism, but based on the observation and analysis, we think the most possible mechanism is by aquatic food, as stated in the result (Line 360-368) and discussion (Line 449-453), which are also supported by the restaurant outbreak. We have now further revised the discussion to make it clear that we think aquatic food was the mostly likely transmission vehicle of L3b and L9 lineages.

Line 449-453: “Foodborne disease outbreaks cause by non-toxigenic *V. cholerae* were reported in many developing and developed countries [16], such as the US Gulf Coast outbreak [57]. **The human infections in Hangzhou are also likely to be foodborne and linked to aquatic food products.** Seafood and aquatic food products contamination with *V. cholerae* have been confirmed in China by an epidemiological investigation, 80% of the isolates belonged to serogroup O1 and O139...”

--------------------

**Editorial and Data Presentation Modifications?**
Use this section for editorial suggestions as well as relatively minor modifications of existing data that would enhance clarity. If the only modifications needed are minor and/or editorial, you may wish to recommend “Minor Revision” or “Accept”. 

Reviewer #1: (No Response)

Reviewer #2: The manuscript was generally well-written and easy to follow. A few small comments:

**(Q28)** 1- The paragraph starting in line 77 is an abrupt transition from the previous paragraph’s discussion of lineages. A better transition to the topic would be helpful.
**Response:** We agree with the reviewer that we need a better transition, we have now revised the text as follows:

Line 76-78: **Despite the high genetic diversity of *V. cholerae*, only lineages encoding the key virulence factors can cause cholera pandemics (e.g. L1 and L2).** Cholera toxin (CT) and toxin co-regulated pilus (TCP) are well-known virulence factors. CT, the protein complex that causes…

**(Q29)** 2- The sentence staring in line 85 is confusing to read. Rewording this sentence would be helpful and could also be used to help transition into this section (see previous comment).
**Response:** We have revised the text to highlight the differences between CT producing and non-toxigenic isolates.

Line 86-98: “**CT producing *V. cholerae* can cause worldwide cholera epidemics, in contrast, non-toxigenic (*ctxAB* negative) isolates tend to be associated with sporadic localized diarrheal disease [16,17].** For instance, non-toxigenic isolates have been…”

**(Q30)** 3- The large arrows in Figure 3a are a bit distracting, and are not necessary to convey the authors’ conclusions.
**Response:** We have removed the arrows in the revised figure.

**(Q31)** 4- It is more common to see “root-to-tip” than “tip-to-root” distance (e.g. line 343 and Fig S5). Perhaps this could be changed for consistency.
**Response:** We have now used “root-to-tip” throughout the manuscript.

**(Q32)** Reviewer #3: Line 237: Please define FSU countries at first use. I would suggest dropping the use of FSU however, and either just state the countries specifically referred to or use the UN or WHO regional grouping names.
**Response:** As suggested by the reviewer, we have removed the word “FSU” and just stated the countries in the revised manuscript.

--------------------

**Summary and General Comments**
Use this section to provide overall comments, discuss strengths/weaknesses of the study, novelty, significance, general execution and scholarship. You may also include additional comments for the author, including concerns about dual publication, research ethics, or publication ethics. If requesting major revision, please articulate the new experiments that are needed.

**(Q33)** Reviewer #1: Comments on manuscript PNTD-D-19-00871 entitled " Genomic epidemiology of Vibrio cholerae reveals the regional and global transmission of two epidemic non-toxigenic lineages"

In this manuscript, the authors describe genomic features of Vibrio cholerae O1 isolates associated with 71 atypical cholera cases in Hangzhou, China between 2001 and 2012. The data generated by this study are interesting as they add knowledge on these atypical non-toxinogenic Vibrio cholerae O1 isolates in terms of clinical symptoms (mild disease) and epidemiology (probable foodborne infection caused by contaminated seafood or aquatic products). Furthermore, this study shows that the MX-2 lineage previously thought to be geographically restricted to Latin America (Domman et al. Science 2017) is globally distributed. 

It would have been, however, interesting to know more on the antimicrobial resistance phenotype and resistome of these Chinese CNTP isolates. Maybe there is no other AMR gene than qnrS. In this case it is worth to be mentioned. For the Chinese isolates containing the qnrS gene within the superintegron (Lineage 3b.4), what is the sensitivity to nalidixic acid and ciprofloxacin?
**Response:** We agree with the reviewer that the antimicrobial resistance information of CNTP isolates is important. we have now tested the resistome of 22-80 CNTP isolates, and found that most of them were antibiotic sensitive, including nalidixic acid and ciprofloxacin (Table S3). We have now included the antimicrobial resistance information in the revised manuscript.

Line 145-152:

**Antimicrobial susceptibility testing**

The antimicrobial susceptibilities of 22-80 randomly selected Hangzhou CNTP isolates were tested using the disk diffusion method on Mueller-Hinton agar according to the guidelines of the Clinical and Laboratory Standards Institute (CLSI, M02-A11). The isolates were categorized as being resistant, intermediate and sensitive to each antimicrobial drug. The susceptibilities to drugs, including ampicillin, amikacin, streptomycin, gentamicin, tetracycline, chloramphenicol, trimethoprim-sulfamethoxazole (SXT) ciprofloxacin, and nalidixic acid, were interpreted (Table S3) using the CLSI criteria (M100-S22) for the *Enterobacteriaceae*.

Line 308: “Virulence factors, **antimicrobial resistance** and unique genomic variants in L3b and L9 isolates”

Line 327-330: The product of *qnrS* gene has been shown to protect gyrase and topoisomerase IV from quinolone action in enteric bacteria [55], however, most of the L3b and L9 lineage isolates are sensitive to commonly used antibiotics (Table S3), including fluoroquinolones (ciprofloxacin and norfloxacin acid).

**(Q34)** Reviewer #2: “Genomic epidemiology of Vibrio cholerae reveals the regional and global transmission of two epidemic non-toxigenic lineages,” describes the analysis of 119 CNTP isolates from Hangzhou. The authors find that these isolates belong to two lineages, and use previously-published cholera sequences to contextualize their transmission on a global scale. They provide evidence for multiple transmission events of these lineages to and from China, and advocate for more widespread surveillance of these non-toxigenic lineages. These findings are well-described with important caveats about sampling, and clearly show that these lineages are being transmitted outside of Latin America — the only place they have been previously reported. With some minor edits, this manuscript could make a nice contribution to the available literature on non-toxigenic lineages, and would add to the growing collection of cholera whole genome sequences. By providing a few more details on how the samples were prepared (see my comment in the Methods section above), the manuscript can also become a resource for others attempting to sequence cholera isolates.
**Response:** We thank the reviewer for the positive feedback, we have now added the description about the sample preparation, see response to comment Q1.

**(Q35)** Reviewer #3: Here the authors have sequenced 119 VC genomes isolated in Hangzhou, China over a period of time stretching between 2001 to 2012. Their results show that many of these non-pandemic strains ‘fill out’ lineages within the larger Vibrio cholerae species tree and will provide a really great resource for future studies. Once we have addressed some of the major concerns I raised above, I would be happy to see this manuscript published.
**Response:** We thank the reviewer for the positive feedback and we have now rectified the concerns.
--------------------

PLOS authors have the option to publish the peer review history of their article ([what does this mean?](https://journals.plos.org/plosntds/s/editorial-and-peer-review-process#loc-peer-review-history)). If published, this will include your full peer review and any attached files.

If you choose “no”, your identity will remain anonymous but your review may still be made public.

**Do you want your identity to be public for this peer review?** For information about this choice, including consent withdrawal, please see our [Privacy Policy](https://www.plos.org/privacy-policy).

Reviewer #1: No

Reviewer #2: No

Reviewer #3: Yes: Daryl Domman
