# Supplementary material for: Genomic epidemiology of Vibrio cholerae reveals the regional and global spread of two epidemic non-toxigenic lineages
Source: PLoS Negl Trop Dis. 2020 Feb 18;14(2):e0008046. doi: 10.1371/journal.pntd.0008046 (PMC7048298; doi:10.1371/journal.pntd.0008046)
Supplement: Attachment — Submitted filename: 20191211-PNTD-R2.VC response Letter_CY_XD.docx [file pntd.0008046.s015.docx]

**Tracking number: PNTD-D-19-00871**

**Title: “Genomic epidemiology of Vibrio cholerae reveals the regional and global spread of two epidemic non-toxigenic lineages”**

**Responses to reviewers’ comments and suggestions:**

**Reviewer's Responses to Questions**

**Key Review Criteria Required for Acceptance?**

As you describe the new analyses required for acceptance, please consider the following:

Methods

-Are the objectives of the study clearly articulated with a clear testable hypothesis stated?

-Is the study design appropriate to address the stated objectives?

-Is the population clearly described and appropriate for the hypothesis being tested?

-Is the sample size sufficient to ensure adequate power to address the hypothesis being tested?

-Were correct statistical analysis used to support conclusions?

-Are there concerns about ethical or regulatory requirements being met?

Reviewer #2: The methods section is much improved in response to the reviewer comments. A few other minor comments to consider:

1- The paragraph starting at line 108 and the paragraph starting at line 136 are largely redundant. Combining these into one clear and concise paragraph would be helpful.

**Response:** We thank the reviewer for the constructive suggestion. we have now combined these two paragraphs into one to remove the redundancy.

2- It would be helpful to add a topic sentence to before line 121 to clarify the purpose of the next two paragraphs (determining which strains are CNTP). They are quite confusing otherwise.

**Response:** We have now revised the text to highlight the purpose as follows:

Line 128-133: To determine the potential gene loss during laboratory culture, the presence/absence of *ctxAB*, *zot*, *ace*, *cep* and *tcpA* were examined by PCR amplification within three days of isolation (most were tested within a day). In a previous study, it has been reported that *ctxAB* genes of *V. cholerae* isolates can be lost in culture, however, the earliest loss took place four days after isolation and the loss was not only the *ctxAB* genes but also the other genes of the CTX prophage [30]. Thus, although the *ctxAB* genes are on a mobile element, the probability of loss during laboratory culturing should be small for our collection.

3- Line 123: It is unclear that you are talking about published studies (timing of ctx loss) and not your own data. Please clarify.

**Response:** we have revised the text to highlight it is a published study as follows:

Line 130-132: In a previous study, it has been reported that *ctxAB* genes of *V. cholerae* isolates can be lost in culture, however, the earliest loss took place four days later and their genomes lost not only the *ctxAB* genes but also the other genes of the CTX prophage [30].

4- Line 156: Why 850 genome sequences? When did you download the sequences and were there only 850 available at the time? Adding the date would be helpful, as there are now over 1200 available.

**Response:** We have added the date for downloading the genomes in the text as follows:

Line 154-155: …and 850 publicly available genome sequences downloaded from NCBI (up to July 2018).

5- Line 205: I agree with Reviewer #3 that the drop from 18000 SNPs to a few hundred after using ClonalFrameML is striking. I appreciate you recombination rate calculations, but are there other software you could use to filter out recombination, so you could compare the results of the two? It's just such a big difference - would be good to check using another method.

**Response:** We have used another software – Gubbins to identify recombination regions of L3b and L9 lineages. The recombination regions identified by the two softwares were highly consistent, nearly all the recombination regions (1.868/1.872 Mb in L3b lineage, 1.065/1.070 Mb in L9 lineage) identified by ClonalFrameML were also found in Gubbins results, further supporting the inference of high recombination rates of these two lineages. We have classified this in the material and method part as follows:

Line 200-204: We also ran Gubbins [40] to identify the recombination regions in the L3b and L9 lineages. The recombination regions identified by the two methods were highly consistent, with almost all the recombination regions (1.868/1.872 Mb in L3b lineage, 1.065/1.070 Mb in L9 lineage) identified by ClonalFrameML being also found by Gubbins, further supporting the inference of high recombination rates of these two lineages.

Reviewer #3: (No Response)

--------------------

Results

-Does the analysis presented match the analysis plan?

-Are the results clearly and completely presented?

-Are the figures (Tables, Images) of sufficient quality for clarity?

Reviewer #2: The results are much more straightforward in the revised manuscript, and I appreciate the toned-down approach to many of the tenuous conclusions.

Line 362: Would it be possible to provide the average number of SNPs between human cases in the various lineages? This would help contextualize the 0-2 SNP number.

**Response:** we have added the average number of SNPs between human cases of L3b and L9 lineages in the text.

Line 365-366: …in contrast, the pairwise SNP distance between human isolates of L3b and L9 lineages were 55 and 127, respectively.

Reviewer #3: Line 46-47: I think the "led to the establishment..." is a bit strong, as we still have only limited sampling of these lineages. Perhaps change to "are related to the groups..."

**Response:** Thank you for the suggestion, we have revised the text accordingly.

Line 52: frame --> framework

**Response:** Thank you for pointing this out, we have corrected the text.

--------------------

Conclusions

-Are the conclusions supported by the data presented?

-Are the limitations of analysis clearly described?

-Do the authors discuss how these data can be helpful to advance our understanding of the topic under study?

-Is public health relevance addressed?

Reviewer #2: (No Response)

Reviewer #3: (No Response)

--------------------

Editorial and Data Presentation Modifications?

Use this section for editorial suggestions as well as relatively minor modifications of existing data that would enhance clarity. If the only modifications needed are minor and/or editorial, you may wish to recommend “Minor Revision” or “Accept”.

Reviewer #2: (No Response)

Reviewer #3: (No Response)

--------------------

Summary and General Comments

Use this section to provide overall comments, discuss strengths/weaknesses of the study, novelty, significance, general execution and scholarship. You may also include additional comments for the author, including concerns about dual publication, research ethics, or publication ethics. If requesting major revision, please articulate the new experiments that are needed.

Reviewer #2: (No Response)

Reviewer #3: I thank the authors for the effort put forth in the revision. All of my comments were addressed and I look forward to seeing this publication in print.

**Response:** We thank the reviewer for your very helpful comments.

--------------------

PLOS authors have the option to publish the peer review history of their article (what does this mean?). If published, this will include your full peer review and any attached files.

If you choose “no”, your identity will remain anonymous but your review may still be made public.

Do you want your identity to be public for this peer review? For information about this choice, including consent withdrawal, please see our Privacy Policy.

Reviewer #2: No

Reviewer #3: No
